# Uncovering the role of USP54 in cancer

**DOI:** 10.18632/oncotarget.14820

**Published:** 2017-01-26

**Authors:** Veronica Miguela, Amaia Lujambio

**Affiliations:** Department of Oncological Sciences and Department of Medicine, Liver Cancer Program, Division of Liver Diseases, Graduate School of Biomedical Sciences at Icahn School of Medicine at Mount Sinai, New York, USA

**Keywords:** deubiquitinases, USP54, colorectal carcinoma, melanoma, therapy

Ubiquitination is a highly dynamic process and can be reverted by deubiquitinases (DUBs), which cleave the isopeptide bond that links ubiquitin to the target proteins [1]. DUBs can also affect ubiquitin maturation, recycling, and editing. There are different types of DUBs, such as ubiquitin-specific proteases (USPs), ubiquitin carboxy-terminal hydrolases (UCHs), Machado-Joseph disease protein domain proteases, JAMM/MPN domain-associated metallopeptidases (JAMMs), monocyte chemotactic protein-induced proteins (MCPIPs), and ovarian-tumor proteases (OTUs) [1]. Among them, the USP class is the most numerous due to a rapid diversification during evolution, possibly in concert with the expansion of E3 ligases [2]. USPs play essential roles in different biological processes, including DNA repair, cell growth, and apoptosis, and are therefore predicted to be involved in cancer [3]. Yet, the contribution of most USPs to tumorigenesis remains unexplored.

In the current issue of *Oncotarget*, Fraile and colleagues (2016)[4] unveil the role of USP54 in promoting cancer, suggesting that USP54 could represent a promising target for anticancer therapies. Fraile *et al.* identify USP54 as overexpressed in intestinal cells with stem cell expression profiles (high levels of *Lgr5* and *EphB2* receptors). Accordingly, silencing of USP54 in colorectal cancer (CRC) cells led to a significant decrease in cell proliferation, colony formation capacity, invasiveness, and tumorigenicity in an *in vivo* xenograft model, indicating that USP54 could promote CRC, potentially by controlling stem cell properties in intestinal cells. To further explore the role of USP54 in CRC, the authors generated USP54-hypomorphic mice, which were then subjected to a CRC-induction protocol. Mice with negligible levels of USP54 showed a decrease in adenocarcinoma formation and in overall colon inflammation when compared to the normal counterparts. Furthermore, the proportion of infiltrating adenocarcinomas and the number of infiltrating tumors per mouse was significantly reduced in USP54-deficient mice, confirming the role of USP54 in CRC initiation and progression. Interestingly, high expression levels of USP54 were significantly associated with poor survival in a cohort of 269 patients with intestinal cancer, further demonstrating the protumorigenic role of USP54.

To investigate the clinical relevance of USP54 beyond CRC, the authors explored publicly available cancer genome databases (cBioPortal, [5]) and found that *USP54* is deleted in malignant peripheral nerve sheath tumors, hinting at a putative tumor suppressor function in this tumor type. In contrast, in neuroendocrine prostate cancer *USP54* undergoes genomic amplification, suggesting a potential oncogenic role. In addition, mutations in this gene are found in colorectal, pancreatic, and endometrial carcinomas as well as in melanoma. These findings prompted the authors to probe the function of USP54 in an *in vivo* melanoma model. Injection of murine melanoma B16F10 cells, with control or USP54-directed short-hairpin RNAs, into recipient mice led to a significant decrease in the number of metastases to the lung, corroborating the pro-metastatic role of USP54 in melanoma progression.

While this study clearly shows the critical role of USP54 in tumorigenesis, several intriguing questions remain unanswered. What is the mechanism behind the oncogenic activity of USP54? What determines the pro-tumorigenic or putative anti-tumorigenic function of USP54 in different malignancies? One possibility is that the role of USP54 in cancer could be linked to the capacity of DUBs to interact with diverse substrates in different cell types [6]. The identification and characterization of such substrates could help elucidate the molecular mechanisms behind the cancer-related activities of USP54. Moreover, the use of the transgenic mice generated in this study could be fundamental to investigate the role of USP54 in other biological processes. For example, the authors find that USP54-hypomorphic female mice accumulate fat mass over time, suggesting that USP54 could exert more intricate functions.

Finally, this study has important therapeutic implications, as USP54 could potentially be a good target for CRC or melanoma. However, it might be difficult to develop effective and specific USP54 inhibitors. One of the challenges is that DUBs from the USP family present a high degree of structural conservation in the active sites of their catalytic domains, which could hinder the specificity of the inhibitors [6]. The use of compounds that simultaneously target several DUBs could lead to an accumulation of polyubiquitinated proteins (for example, misfolded proteins labeled for degradation) and a decrease in the pool of free monomeric ubiquitin, disrupting the fine tuned balance of ubiquitin within the cell [7]. Such phenomenon could induce proteotoxicity and ultimately cell death, which is interesting regarding cancer cells but could also affect the surrounding healthy tissues. One alternative to avoid cross-reactivity and enhance specificity would be to design compounds targeting other domains involved in substrate recognition or protein interaction, which harbor less degree of sequence and/or structure conservation [6]. Future work will undoubtedly address this and other issues and continue to uncover the role of USP54 and DUBs in cancer and other biological settings.

## References

[1] Komander D (2009). Nat Rev Mol Cell Biol.

[2] Semple CA (2003). Genome Res.

[3] Mason SD (2011). Trends Cell Biol.

[4] Fraile JM (2016). Oncotarget.

[5] Cerami E (2012). Cancer Discov.

[6] Pfoh R (2015). Endocr Relat Cancer.

[7] D'Arcy P (2015). Pharmacology & therapeutics.

